# 3-(2,4-Dichloro­benzyl­idene)-1,5-dioxa­spiro­[5.5]undecane-2,4-dione

**DOI:** 10.1107/S1600536811016813

**Published:** 2011-05-07

**Authors:** Wu-Lan Zeng

**Affiliations:** a MicroScale Science Institute, Department of Chemistry and Chemical Engineering, Weifang University, Weifang 261061, People’s Republic of China

## Abstract

In the title mol­ecule, C_16_H_14_Cl_2_O_4_, the 1,3-dioxane and cyclo­hexane rings exhibit distorted boat and chair conformations, respectively. In the crystal, a pair of weak inter­molecular C—H⋯O hydrogen bonds link the mol­ecules into an inversion dimer.

## Related literature

For ring puckering parameters, see: Cremer & Pople (1975 ▶). For related structures, see: Zeng (2011*a* ▶,*b* ▶).
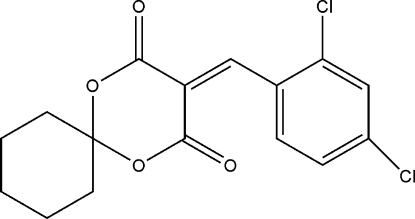


## Experimental

### 

#### Crystal data

C_16_H_14_Cl_2_O_4_
*M* *_r_* = 341.17
Triclinic, 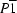

*a* = 7.2378 (6) Å
*b* = 7.6496 (7) Å
*c* = 14.8099 (13) Å
α = 100.569 (2)°
β = 100.870 (2)°
γ = 99.050 (1)°
*V* = 775.80 (12) Å^3^
*Z* = 2
Mo *K*α radiation
μ = 0.43 mm^−1^
*T* = 298 K
0.24 × 0.22 × 0.16 mm

#### Data collection

Bruker SMART CCD area-detector diffractometer
Absorption correction: multi-scan (*SADABS*; Sheldrick, 1996 ▶) *T*_min_ = 0.903, *T*_max_ = 0.934
4085 measured reflections
2697 independent reflections
1596 reflections with *I* > 2σ(*I*)
*R*_int_ = 0.023

#### Refinement

*R*[*F* ^2^ > 2σ(*F* ^2^)] = 0.040
*wR*(*F* ^2^) = 0.073
*S* = 1.01
2697 reflections
199 parameters
H-atom parameters constrained
Δρ_max_ = 0.19 e Å^−3^
Δρ_min_ = −0.21 e Å^−3^

### 

Data collection: *SMART* (Bruker, 1997 ▶); cell refinement: *SAINT* (Bruker, 1997 ▶); data reduction: *SAINT*; program(s) used to solve structure: *SHELXS97* (Sheldrick, 2008 ▶); program(s) used to refine structure: *SHELXL97* (Sheldrick, 2008 ▶); molecular graphics: *SHELXTL* (Sheldrick, 2008 ▶); software used to prepare material for publication: *SHELXTL*.

## Supplementary Material

Crystal structure: contains datablocks global, I. DOI: 10.1107/S1600536811016813/is2706sup1.cif

Structure factors: contains datablocks I. DOI: 10.1107/S1600536811016813/is2706Isup2.hkl

Supplementary material file. DOI: 10.1107/S1600536811016813/is2706Isup3.cml

Additional supplementary materials:  crystallographic information; 3D view; checkCIF report

## Figures and Tables

**Table 1:** Hydrogen-bond geometry (Å, °)

*D*—H⋯*A*	*D*—H	H⋯*A*	*D*⋯*A*	*D*—H⋯*A*
C13—H13⋯O3^i^	0.93	2.50	3.286 (3)	143

Symmetry code: (i) 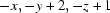.

**Fig. 1.**
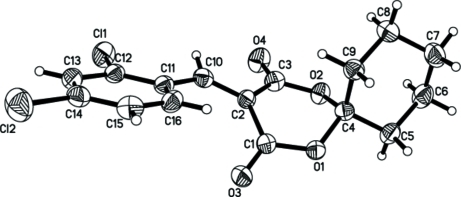
The molecular structure of the title compound, drawn with 30% probability ellipsoids and spheres of arbitrary size for the H atoms.

## supplementary crystallographic information

### Comment

We have recently reported the crystal structures of 3-(4-bromobenzylidene)-1,5-dioxaspiro[5.5]undecane-2,4-dione and 3-(4-fluorobenzylidene)-1,5-dioxaspiro[5.5]undecane-2,4-dione (Zeng, 2011*a*,*b*). As part of our ongoing studies on new spiro compounds with potentially higher bioactivity, the title compound, (I), has been synthesized.

The crystal structure analysis (Fig. 1) confirms that the 1,3-dioxane ring and the benzene ring are connected *via* C10-C11 single bond [1.462 (3) Å] and C2═C10 double bond [1.346 (3) Å]. The 1,3-dioxane ring has a distorted boat conformation with C4 atom common to the cyclohexane forming the flap. The cyclohexane ring exists in a chair conformation, with puckering parameters (Cremer & Pople, 1975) Q = 0.552 Å, θ = 0.7°, φ = 242.54°.

### Experimental

The mixture of malonic acid (6.24 g, 0.06 mol) and acetic anhydride (9 ml) in strong sulfuric acid (0.25 ml) was stirred with water at 303K, After dissolving, cyclohexanone (5.88 g, 0.06 mol) was added dropwise into solution for 1 h. The reaction was allowed to proceed for 4 h. The mixture was cooled and filtered, and then an ethanol solution of 2,4-dichlorobenzaldehyde (10.44 g, 0.06 mol) was added. The solution was then filtered and concentrated. Single crystals were obtained by evaporation of an ethanol solution of (I) at room temperature over a period of one week.

### Refinement

H atoms were placed in calculated positions (C—H = 0.93 or 0.97 Å) and refined as riding, with *U*_iso_(H) = 1.2*U*_eq_(C).

### Crystal data

**Table:**

C_16_H_14_Cl_2_O_4_	*Z* = 2
*M**_r_* = 341.17	*F*(000) = 352
Triclinic, *P*1	*D*_x_ = 1.461 Mg m^−^^3^
Hall symbol: -P 1	Mo *K*α radiation, λ = 0.71073 Å
*a* = 7.2378 (6) Å	Cell parameters from 1133 reflections
*b* = 7.6496 (7) Å	θ = 3.0–26.3°
*c* = 14.8099 (13) Å	µ = 0.43 mm^−^^1^
α = 100.569 (2)°	*T* = 298 K
β = 100.870 (2)°	Block, colorless
γ = 99.050 (1)°	0.24 × 0.22 × 0.16 mm
*V* = 775.80 (12) Å^3^	

### Data collection

**Table:**

Bruker SMART CCD area-detector diffractometer	2697 independent reflections
Radiation source: fine-focus sealed tube	1596 reflections with *I* > 2σ(*I*)
graphite	*R*_int_ = 0.023
φ and ω scans	θ_max_ = 25.0°, θ_min_ = 2.8°
Absorption correction: multi-scan (*SADABS*; Sheldrick, 1996)	*h* = −8→7
*T*_min_ = 0.903, *T*_max_ = 0.934	*k* = −9→8
4085 measured reflections	*l* = −15→17

### Refinement

**Table:**

Refinement on *F*^2^	Primary atom site location: structure-invariant direct methods
Least-squares matrix: full	Secondary atom site location: difference Fourier map
*R*[*F*^2^ > 2σ(*F*^2^)] = 0.040	Hydrogen site location: inferred from neighbouring sites
*wR*(*F*^2^) = 0.073	H-atom parameters constrained
*S* = 1.01	*w* = 1/[σ^2^(*F*_o_^2^) + (0.0188*P*)^2^] where *P* = (*F*_o_^2^ + 2*F*_c_^2^)/3
2697 reflections	(Δ/σ)_max_ = 0.001
199 parameters	Δρ_max_ = 0.19 e Å^−^^3^
0 restraints	Δρ_min_ = −0.21 e Å^−^^3^

### Special details

**Table:**

Geometry. All e.s.d.'s (except the e.s.d. in the dihedral angle between two l.s. planes) are estimated using the full covariance matrix. The cell e.s.d.'s are taken into account individually in the estimation of e.s.d.'s in distances, angles and torsion angles; correlations between e.s.d.'s in cell parameters are only used when they are defined by crystal symmetry. An approximate (isotropic) treatment of cell e.s.d.'s is used for estimating e.s.d.'s involving l.s. planes.
Refinement. Refinement of *F*^2^ against ALL reflections. The weighted *R*-factor *wR* and goodness of fit *S* are based on *F*^2^, conventional *R*-factors *R* are based on *F*, with *F* set to zero for negative *F*^2^. The threshold expression of *F*^2^ > σ(*F*^2^) is used only for calculating *R*-factors(gt) *etc*. and is not relevant to the choice of reflections for refinement. *R*-factors based on *F*^2^ are statistically about twice as large as those based on *F*, and *R*- factors based on ALL data will be even larger.

### Fractional atomic coordinates and isotropic or equivalent isotropic displacement parameters (Å^2^)

**Table:**

	*x*	*y*	*z*	*U*_iso_*/*U*_eq_	
Cl1	0.24503 (11)	1.26534 (9)	0.42676 (5)	0.0765 (3)	
Cl2	0.26105 (12)	1.05072 (12)	0.75170 (5)	0.0981 (3)	
O1	0.0537 (2)	0.4031 (2)	0.21259 (10)	0.0524 (4)	
O2	0.1859 (2)	0.5715 (2)	0.11368 (10)	0.0544 (4)	
O3	−0.0445 (2)	0.5412 (2)	0.33392 (12)	0.0641 (5)	
O4	0.2444 (3)	0.8711 (2)	0.14747 (12)	0.0737 (6)	
C1	0.0566 (4)	0.5535 (3)	0.27859 (17)	0.0477 (6)	
C2	0.1771 (3)	0.7246 (3)	0.27024 (16)	0.0446 (6)	
C3	0.2092 (4)	0.7332 (4)	0.17488 (18)	0.0542 (7)	
C4	0.1872 (3)	0.4094 (3)	0.15131 (15)	0.0475 (6)	
C5	0.1083 (4)	0.2509 (3)	0.06858 (15)	0.0590 (7)	
H5A	−0.0152	0.2648	0.0348	0.071*	
H5B	0.0890	0.1396	0.0911	0.071*	
C6	0.2453 (5)	0.2383 (4)	0.00165 (18)	0.0758 (9)	
H6A	0.1968	0.1292	−0.0477	0.091*	
H6B	0.2497	0.3416	−0.0278	0.091*	
C7	0.4462 (5)	0.2341 (4)	0.0524 (2)	0.0789 (9)	
H7A	0.5305	0.2360	0.0087	0.095*	
H7B	0.4447	0.1222	0.0745	0.095*	
C8	0.5242 (4)	0.3952 (4)	0.13604 (18)	0.0693 (8)	
H8A	0.5420	0.5064	0.1133	0.083*	
H8B	0.6483	0.3829	0.1699	0.083*	
C9	0.3869 (3)	0.4063 (3)	0.20302 (16)	0.0540 (7)	
H9A	0.3826	0.3025	0.2322	0.065*	
H9B	0.4343	0.5152	0.2525	0.065*	
C10	0.2437 (3)	0.8767 (3)	0.33824 (16)	0.0504 (6)	
H10	0.2987	0.9759	0.3175	0.060*	
C11	0.2451 (3)	0.9140 (3)	0.43884 (16)	0.0466 (6)	
C12	0.2502 (3)	1.0907 (3)	0.48706 (18)	0.0499 (6)	
C13	0.2546 (3)	1.1339 (3)	0.58226 (18)	0.0583 (7)	
H13	0.2557	1.2523	0.6122	0.070*	
C14	0.2571 (4)	0.9983 (4)	0.63227 (17)	0.0614 (7)	
C15	0.2579 (4)	0.8223 (4)	0.58863 (18)	0.0626 (7)	
H15	0.2625	0.7323	0.6231	0.075*	
C16	0.2516 (3)	0.7824 (3)	0.49329 (17)	0.0556 (7)	
H16	0.2518	0.6639	0.4641	0.067*	

### Atomic displacement parameters (Å^2^)

**Table:**

	*U*^11^	*U*^22^	*U*^33^	*U*^12^	*U*^13^	*U*^23^
Cl1	0.0930 (6)	0.0474 (4)	0.0950 (5)	0.0228 (4)	0.0231 (4)	0.0214 (4)
Cl2	0.1014 (7)	0.1296 (8)	0.0606 (5)	0.0288 (6)	0.0176 (4)	0.0099 (5)
O1	0.0548 (12)	0.0464 (10)	0.0572 (10)	0.0053 (8)	0.0212 (9)	0.0094 (8)
O2	0.0702 (13)	0.0456 (10)	0.0496 (9)	0.0117 (9)	0.0128 (9)	0.0161 (9)
O3	0.0681 (13)	0.0590 (12)	0.0697 (12)	0.0082 (10)	0.0341 (11)	0.0100 (10)
O4	0.1040 (17)	0.0512 (12)	0.0730 (13)	0.0128 (11)	0.0244 (11)	0.0288 (10)
C1	0.0491 (17)	0.0479 (16)	0.0474 (15)	0.0122 (13)	0.0102 (13)	0.0121 (13)
C2	0.0489 (16)	0.0360 (14)	0.0520 (15)	0.0104 (12)	0.0141 (13)	0.0126 (12)
C3	0.0559 (18)	0.0515 (17)	0.0574 (17)	0.0132 (14)	0.0092 (14)	0.0189 (15)
C4	0.0528 (17)	0.0466 (15)	0.0479 (14)	0.0090 (13)	0.0178 (13)	0.0166 (12)
C5	0.070 (2)	0.0499 (16)	0.0520 (15)	0.0002 (14)	0.0152 (14)	0.0059 (13)
C6	0.103 (3)	0.0626 (19)	0.0573 (17)	−0.0016 (18)	0.0341 (18)	0.0017 (15)
C7	0.088 (3)	0.072 (2)	0.090 (2)	0.0206 (18)	0.051 (2)	0.0148 (18)
C8	0.059 (2)	0.0699 (19)	0.0822 (19)	0.0148 (16)	0.0238 (16)	0.0149 (17)
C9	0.0538 (18)	0.0544 (16)	0.0552 (15)	0.0125 (13)	0.0128 (14)	0.0135 (13)
C10	0.0478 (17)	0.0459 (16)	0.0642 (17)	0.0165 (13)	0.0163 (13)	0.0191 (14)
C11	0.0400 (16)	0.0424 (15)	0.0575 (16)	0.0100 (12)	0.0089 (12)	0.0117 (13)
C12	0.0423 (16)	0.0448 (15)	0.0614 (16)	0.0100 (12)	0.0090 (12)	0.0103 (13)
C13	0.0466 (17)	0.0541 (17)	0.0687 (18)	0.0129 (13)	0.0097 (14)	0.0008 (15)
C14	0.0440 (18)	0.076 (2)	0.0596 (17)	0.0110 (15)	0.0103 (13)	0.0060 (16)
C15	0.0577 (19)	0.070 (2)	0.0611 (18)	0.0108 (15)	0.0078 (15)	0.0236 (16)
C16	0.0557 (18)	0.0443 (15)	0.0623 (17)	0.0084 (13)	0.0069 (14)	0.0086 (14)

### Geometric parameters (Å, °)

**Table:**

Cl1—C12	1.739 (2)	C7—C8	1.526 (3)
Cl2—C14	1.734 (2)	C7—H7A	0.9700
O1—C1	1.361 (3)	C7—H7B	0.9700
O1—C4	1.446 (2)	C8—C9	1.532 (3)
O2—C3	1.358 (3)	C8—H8A	0.9700
O2—C4	1.450 (2)	C8—H8B	0.9700
O3—C1	1.203 (2)	C9—H9A	0.9700
O4—C3	1.206 (3)	C9—H9B	0.9700
C1—C2	1.493 (3)	C10—C11	1.462 (3)
C2—C10	1.346 (3)	C10—H10	0.9300
C2—C3	1.485 (3)	C11—C12	1.400 (3)
C4—C5	1.507 (3)	C11—C16	1.402 (3)
C4—C9	1.509 (3)	C12—C13	1.381 (3)
C5—C6	1.530 (3)	C13—C14	1.382 (3)
C5—H5A	0.9700	C13—H13	0.9300
C5—H5B	0.9700	C14—C15	1.383 (3)
C6—C7	1.514 (4)	C15—C16	1.379 (3)
C6—H6A	0.9700	C15—H15	0.9300
C6—H6B	0.9700	C16—H16	0.9300
			
C1—O1—C4	119.82 (19)	H7A—C7—H7B	108.0
C3—O2—C4	118.34 (17)	C7—C8—C9	111.0 (2)
O3—C1—O1	118.7 (2)	C7—C8—H8A	109.4
O3—C1—C2	125.7 (2)	C9—C8—H8A	109.4
O1—C1—C2	115.4 (2)	C7—C8—H8B	109.4
C10—C2—C3	117.0 (2)	C9—C8—H8B	109.4
C10—C2—C1	126.3 (2)	H8A—C8—H8B	108.0
C3—C2—C1	116.4 (2)	C4—C9—C8	111.23 (19)
O4—C3—O2	118.8 (2)	C4—C9—H9A	109.4
O4—C3—C2	124.9 (2)	C8—C9—H9A	109.4
O2—C3—C2	116.2 (2)	C4—C9—H9B	109.4
O1—C4—O2	108.91 (17)	C8—C9—H9B	109.4
O1—C4—C5	106.67 (19)	H9A—C9—H9B	108.0
O2—C4—C5	106.30 (18)	C2—C10—C11	131.6 (2)
O1—C4—C9	111.22 (17)	C2—C10—H10	114.2
O2—C4—C9	110.75 (19)	C11—C10—H10	114.2
C5—C4—C9	112.76 (19)	C12—C11—C16	116.3 (2)
C4—C5—C6	111.0 (2)	C12—C11—C10	120.1 (2)
C4—C5—H5A	109.4	C16—C11—C10	123.5 (2)
C6—C5—H5A	109.4	C13—C12—C11	122.5 (2)
C4—C5—H5B	109.4	C13—C12—Cl1	117.25 (19)
C6—C5—H5B	109.4	C11—C12—Cl1	120.21 (19)
H5A—C5—H5B	108.0	C12—C13—C14	118.8 (2)
C7—C6—C5	111.9 (2)	C12—C13—H13	120.6
C7—C6—H6A	109.2	C14—C13—H13	120.6
C5—C6—H6A	109.2	C13—C14—C15	121.0 (2)
C7—C6—H6B	109.2	C13—C14—Cl2	119.2 (2)
C5—C6—H6B	109.2	C15—C14—Cl2	119.8 (2)
H6A—C6—H6B	107.9	C16—C15—C14	119.0 (2)
C6—C7—C8	111.6 (2)	C16—C15—H15	120.5
C6—C7—H7A	109.3	C14—C15—H15	120.5
C8—C7—H7A	109.3	C15—C16—C11	122.3 (2)
C6—C7—H7B	109.3	C15—C16—H16	118.8
C8—C7—H7B	109.3	C11—C16—H16	118.8
			
C4—O1—C1—O3	−173.9 (2)	C6—C7—C8—C9	54.4 (3)
C4—O1—C1—C2	10.6 (3)	O1—C4—C9—C8	174.74 (18)
O3—C1—C2—C10	23.5 (4)	O2—C4—C9—C8	−64.0 (2)
O1—C1—C2—C10	−161.3 (2)	C5—C4—C9—C8	55.0 (3)
O3—C1—C2—C3	−149.8 (2)	C7—C8—C9—C4	−54.3 (3)
O1—C1—C2—C3	25.3 (3)	C3—C2—C10—C11	−176.9 (2)
C4—O2—C3—O4	166.2 (2)	C1—C2—C10—C11	9.8 (4)
C4—O2—C3—C2	−17.3 (3)	C2—C10—C11—C12	−154.4 (2)
C10—C2—C3—O4	−19.7 (4)	C2—C10—C11—C16	29.0 (4)
C1—C2—C3—O4	154.3 (2)	C16—C11—C12—C13	−2.2 (4)
C10—C2—C3—O2	164.1 (2)	C10—C11—C12—C13	−179.0 (2)
C1—C2—C3—O2	−21.9 (3)	C16—C11—C12—Cl1	179.42 (17)
C1—O1—C4—O2	−47.4 (2)	C10—C11—C12—Cl1	2.6 (3)
C1—O1—C4—C5	−161.76 (18)	C11—C12—C13—C14	1.1 (4)
C1—O1—C4—C9	74.9 (2)	Cl1—C12—C13—C14	179.48 (19)
C3—O2—C4—O1	51.0 (3)	C12—C13—C14—C15	0.8 (4)
C3—O2—C4—C5	165.5 (2)	C12—C13—C14—Cl2	−179.59 (19)
C3—O2—C4—C9	−71.6 (2)	C13—C14—C15—C16	−1.4 (4)
O1—C4—C5—C6	−176.71 (19)	Cl2—C14—C15—C16	178.97 (19)
O2—C4—C5—C6	67.2 (3)	C14—C15—C16—C11	0.2 (4)
C9—C4—C5—C6	−54.3 (3)	C12—C11—C16—C15	1.6 (4)
C4—C5—C6—C7	53.8 (3)	C10—C11—C16—C15	178.3 (2)
C5—C6—C7—C8	−54.4 (3)		

### Hydrogen-bond geometry (Å, °)

**Table:**

*D*—H···*A*	*D*—H	H···*A*	*D*···*A*	*D*—H···*A*
C13—H13···O3^i^	0.93	2.50	3.286 (3)	143

Symmetry codes: (i) −*x*, −*y*+2, −*z*+1.
